# Functionalized Biopolymer for Enhanced Pt(IV) Recovery from Aqueous Solutions

**DOI:** 10.3390/polym17091132

**Published:** 2025-04-22

**Authors:** Theodora Babău, Mihaela Ciopec, Giannin Mosoarca, Cosmin Vancea, Adina Negrea, Nicoleta Sorina Nemeş, Bogdan Pascu, Petru Negrea, Catalin Ianăşi, Alina Ramona Buzatu

**Affiliations:** 1 Faculty of Chemical Engineering, Biotechnologies and Environmental Protection, Politehnica University Timisoara, Victoriei Square, no. 2, 300006 Timisoara, Romania; theodora.babau@student.upt.ro (T.B.); mihaela.ciopec@upt.ro (M.C.); adina.negrea@upt.ro (A.N.); petru.negrea@upt.ro (P.N.); 2 Renewable Energy Research Institute-ICER, Politehnica University Timişoara, Gavril Musicescu Street, no. 138, 300774 Timisoara, Romania; nicoleta.nemes@upt.ro (N.S.N.); bogdan.pascu@upt.ro (B.P.); 3 ISIM-National R&D Institute for Welding and Material Testing, Timisoara, Bv. Mihai Viteazu, nr. 30, 300222 Timisoara, Romania; 4 “Coriolan Drăgulescu” Institute of Chemistry, Romanian Academy, Bv. Mihai Viteazu, No. 24, 300223 Timisoara, Romania; ianasic@acad-icht.tm.edu.ro; 5 Faculty of Medicine, “Victor Babes” University of Medicine and Pharmacy, Eftimie Murgu Square, no. 2, 300041 Timisoara, Romania; buzatu.ramona@umft.ro

**Keywords:** platinum, chitosan, serine, adsorption, recovery, batch adsorption, column adsorption

## Abstract

In this study, chitosan (Chi) functionalized with the amino acid serine (Ser) was synthesized for the adsorption-based recovery of Pt(IV) from aqueous solutions. To identify the active functional groups of the amino acid and the support material, the synthesized adsorbent was characterized using SEM, FT-IR, and EDX analyses, and its point of zero charge (pH_PZC_) was determined. Static and dynamic adsorption studies were conducted to optimize process parameters. Under static conditions, equilibrium studies established the maximum Pt(IV) concentration that could be adsorbed onto Chi–Ser, as well as its maximum adsorption capacity. At pH > 4, with an S-L ratio of 0.1 g:25 mL Pt(IV) solution, a contact time of 90 min, and a temperature of 298 K, the maximum adsorption capacity reached 7.23 mg/g. The adsorption process was best described by the Sips isotherm. The Taguchi method was employed to optimize static adsorption conditions. The Clark equation most accurately modeled the adsorption process under dynamic conditions. Additionally, multiple adsorption–desorption cycles evaluated the adsorbent’s reusability.

## 1. Introduction

The intensive mining of platinum group metals (PGMs) has significantly depleted global reserves [1].

PGMs are strategically important to many industries, making their recovery essential for maintaining a stable supply chain, reducing import dependency, and minimizing the need for further mining—thereby mitigating environmental impact [2,3,4,5]. The choice of Pt(IV) recovery technology depends on the material source (e-waste, automotive catalysts, jewelry, etc.) and metal concentration [6].

Common approaches for the recovery of platinum group metals (PGMs) include the following: (i) hydrometallurgy, which involves dissolving metals in a strong aqueous solution [7,8], followed by separation through solvent extraction [9,10] and subsequent precipitation as salts or pure metals [11,12,13]; (ii) pyrometallurgy, wherein PGMs are extracted by melting materials at elevated temperatures [14,15,16,17]; (iii) biohydrometallurgy, which utilizes microorganisms such as bacteria and fungi to oxidize or solubilize PGM-bearing substrates [18,19,20]; and (iv) physical separation techniques, including grinding, flotation, and magnetic separation [1]. Despite their effectiveness, these methods are often associated with high operational costs, substantial energy requirements, and the potential for secondary environmental pollution [21].

Adsorption is a highly efficient physicochemical process in which platinum ions bind to the surface of an adsorbent. Owing to its selectivity, versatility, and effectiveness across varying concentrations, it is increasingly employed for platinum and other precious metal recovery.

Common platinum adsorbents include ion exchange resins, activated carbon, nanocomposites, and functionalized organic materials with electron-donating groups that form stable complexes with platinum ions [22,23,24,25,26]. Recently, biopolymers such as chitosan—a biodegradable, non-toxic, and biocompatible poly(D-glucosamine) derived from chitin—have emerged as sustainable alternatives due to their environmental friendliness and wide applicability [27].

Chitosan is a well-studied adsorbent for metal ions, dyes, and proteins, with its hydroxyl and amino groups enabling stable chelate formation with transition metals [28]. Its higher amino group content gives it superior adsorption capacity compared to chitin [29]. To enhance metal ion selectivity, various functional groups—such as poly(ethyleneimine) [30], thiourea [31], and rubeanic acid derivatives [32,33]—have been grafted onto chitosan via glutaraldehyde linkage. Selectivity depends on the complexing agent, with N- and S-containing ligands showing high affinity for precious metals. Functionalization with ligands like 3,4-diaminobenzoic acid [34], N-methyl-D-glucamine [35], ethylenediamine [36], and 2[-bis-(pyridylmethyl)aminomethyl]-4-methyl-6-formylphenol [37] has been reported, though serine-crosslinked chitosan has not yet been explored.

In this study, chitosan (Chi) functionalized with the amino acid serine (Ser) was synthesized and used for the adsorption-based recovery of Pt(IV) from aqueous solutions. To identify the active functional groups of both the amino acid and the chitosan support, the synthesized material was characterized using scanning electron microscopy (SEM), infrared spectroscopy (FT-IR), and energy-dispersive X-Ray spectroscopy (EDX).

Static and dynamic adsorption studies were conducted to determine key process parameters, as well as the isotherms and kinetic models that best describe the adsorption behavior. The Taguchi method was employed to optimize adsorption conditions in a batch system. Finally, the reusability of the adsorbent material was evaluated through multiple adsorption–desorption cycles.

## 2. Materials and Methods

### 2.1. Synthesis of the Chi–Ser Material

This study set an ambitious and highly relevant objective in the current economic and political context: developing new adsorbent materials for the recovery of platinum group metal ions. To achieve this, 25 materials were synthesized using chitosan as a solid support and various amino acids as extractants.

The selection of chitosan (Chi) as the support material is well-founded, given its natural abundance, low cost, and intrinsic adsorptive properties. Likewise, the choice of amino acids as extractants is justified by their functional –NH_2_ (amino) and –COOH (carboxyl) groups, which provide exceptional versatility in interacting with different compounds. The amino group can act as a base, forming hydrogen bonds or ionic interactions, while the carboxyl group behaves as an acid, also participating in hydrogen bonding and ionic interactions. This dual functionality makes amino acids excellent metal chelators.

Additionally, amino acids exhibit amphoteric behavior, meaning they can function as both acids and bases, allowing them to adapt to varying pH conditions and interact with a wide range of substances. Their biodegradability further enhances their appeal, as they are naturally occurring compounds that minimize the environmental impact of the extraction process. Moreover, amino acids are readily available at relatively low costs, making them suitable for large-scale applications.

Thus, aspartic acid (Asp) and L-glutamic acid (Glu) have two carboxyl groups and can form stable chelates with bivalent and trivalent metals. Valine (Val), although it has a more hydrophobic structure than the other chosen amino acids, can contribute to hydrophobic interactions with organic molecules. DL-cysteine (Cys) has a sulfhydryl group (-SH), which gives it reducing properties and a special affinity for heavy metals. Serine (Ser) has a hydroxyl group (-OH) that can form hydrogen bonds and contribute to polar interactions with other molecules [27,38]. All amino acids and hexachloroplatinic acid (H_2_PtCl_6_) were obtained from Merck & Co., Inc. (Rahway, NJ, USA) and possessed analytical grade purity (99.9%). Chitosan (40 mesh; degree of deacetylation—90%) was supplied by Yuhuan Ocean Biology Co., Ltd. (Yuhuan, China).

The proposed procedure employs a rigorous ultrasonication method for the functionalization of chitosan with the selected amino acids. The use of ultrasound in this process offers several key advantages: acceleration of the dissolution and diffusion process; ultrasound producing cavities that improve mass transfer and favor the interaction between the support and the extractant; improvement in the homogeneity of the mixture; ensuring uniform dispersion of the extractant in suspension by creating micro-turbulences; and reduction of the reaction time compared to conventional methods, which allows obtaining similar results in a shorter time.

A total of 25 materials were synthesized by functionalizing chitosan (Chi) via ultrasonication with five different amino acids—aspartic acid (Asp), L-glutamic acid (Glu), valine (Val), DL-cysteine (Cys), and serine (Ser)—at five distinct chitosan-to-amino acid mass ratios (1:0.05, 1:0.10, 1:0.15, 1:0.20, and 1:0.25). These materials were evaluated as adsorbents for Pt(IV) recovery under the following conditions: initial Pt(IV) concentration (C_0_) = 10 mg/L, contact time = 1 h, and temperature = 298 K. The adsorption capacity was subsequently determined. Among the tested materials, the chitosan functionalized with serine exhibited the highest adsorption capacity (2.05–2.25 mg/g). The selected mass ratio for Chi–Ser material synthesis was 1:0.1, as it provided a relatively high adsorption capacity, while higher ratios were not economically justified.

The amino acids were first dissolved in 25 mL of deionized water and subsequently introduced to the chitosan. The resulting mixture was subjected to ultrasonic treatment using an ultrasonic bath (Sonorex Super 10 P, Bandelin electronic GmbH & Co. KG, Berlin, Germany) for 10 min at a temperature of 298 K, operating at a frequency of 50 Hz and a power range of 160–480 W.

**Figure 1:**
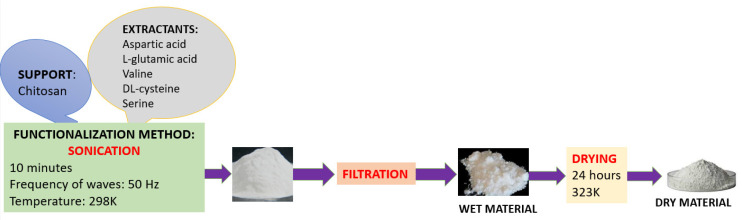
Schematic of the materials synthesis process.

A schematic representation of the material synthesis process is shown in Figure 1.

### 2.2. Adsorbents Testing for Pt(IV) Recovery

By comparing the adsorption capacity of the 25 synthesized materials, the most effective material for Pt(IV) recovery was identified. The test conditions were pH > 4, temperature 298 K, and contact time 60 min. Approximately 0.1 g of adsorbent material was weighed, to which 25 mL of the 10 mg/L Pt(IV) solution was added. The studies were carried out in a thermostated and stirred bath with a rotation speed of 200 rpm (Julabo, SW23, Julabo GmbH, Seelbach, Germany). The concentration of Pt(IV) in the aqueous solutions was precisely determined using atomic absorption spectrometry (AAS, Varian SpectrAA 280 FS, Varian Inc., Mulgrave, Australia), a highly sensitive and reliable technique for this type of analysis.

The adsorption capacity was calculated using Equation (1):

(1) $$q=\frac{(Ci-Crez)\cdot V}{m}$$

where *Ci*—initial concentration of Pt(IV), mg/L; *Crez*—residual concentration of Pt(IV), mg/L; *V*—solution volume, L; and *m*—mass of adsorbent, g.

The selected material, Chi–Ser, chosen for its superior affinity for Pt(IV) was characterized physicochemically by scanning electron spectroscopy (SEM) and energy-dispersive X-Ray spectroscopy (EDX) using a Quanta FEG 250 microscope (FEI, Hilsboro, OR, USA) and infrared spectroscopy (FT-IR) using JASCO FT/IR-4200 apparatus (SpectraLab, Shimadzu, Japan).

The pH_pZc_ of the Chi–Ser material was determined [39]. A 0.1 g sample of Chi–Ser adsorbent material was suspended in 25 mL of 0.01 M KCl solution and maintained under continuous stirring at 200 rpm and 298 K for 60 min in a thermostated water bath.

The pH of the KCl solutions was adjusted within the 1–10 range using NaOH (0.05 N–2 N) or HNO_3_ (0.05 N–2 N) solutions. After the contact period, the final pH (pHf) of the filtered samples was measured using a Mettler Toledo Seven Compact S210 pH meter.

To determine the point of zero charge (pH_pZc_) of the adsorbent material, the final pH (pHf) was plotted against the initial pH (pHi).

### 2.3. Pt(IV) Recovery Studies Batch Adsorption Experiments

Optimizing the adsorption process is crucial and represents a robust scientific approach for developing efficient adsorbent materials for Pt(IV) recovery from aqueous solutions. Thus, valuable information was obtained by studying the influence of the S:L ratio, pH, contact time, and initial Pt(IV) concentration on process efficiency and adsorption capacity.

A higher S-L ratio provides a larger contact surface between the adsorbent and the solution, which favors adsorption. However, an excessively high ratio may result in rapid saturation of the adsorbent, leading to inefficient material utilization.

Experiments were conducted at various S-L ratios to identify the optimal value for maximum adsorption efficiency. At pH > 4, 60 min of contact time, and 298 K, different amounts of Chi–Ser material (0.025, 0.05, 0.075, 0.1, 0.15, 0.2, and 0.3 g) were weighed and mixed with 25 mL of 10 mg/L Pt(IV) solution. The samples were then stirred in a thermostatic water bath at 298 K and 200 rpm for 60 min.

The pH was adjusted using 0.05–2 N NaOH and 0.05–2 N HNO₃ solutions, with the Pt(IV) solution prepared at a concentration of 10 mg/L. A 0.1 g sample of Chi–Ser material was weighed and mixed with 25 mL of Pt(IV) solution at the desired pH (ranging from 1 to 10). The samples were then placed in a thermostatic and stirred water bath for 60 min at 298 K, after which they were filtered.

To investigate the effect of contact time on the adsorption capacity of Chi–Ser, 0.1 g of material was weighed, and 25 mL of 10 mg/L Pt(IV) solution (with pH > 4) was added. The material was kept in contact with the solution for varying durations (15, 30, 45, 60, 90, and 120 min) in a thermostatic and stirred water bath at 200 rpm. After the specified contact times, the samples were filtered.

To determine the maximum initial Pt(IV) concentration that Chi–Ser material can retain under the previously established optimal adsorption conditions, the initial concentrations of Pt(IV) solutions were varied between 5 and 80 mg/L. The experiments were conducted with 60 min contact time, 298 K temperature, and pH > 4.

The residual Pt(IV) concentration was measured using atomic absorption spectrometry (AAS) with a Varian SpectrAA 280 FS spectrophotometer (Varian Inc., Mulgrave, Australia).

The Langmuir, Freundlich, and Sips isotherms are the most commonly used models for describing adsorption processes. Each model provides unique insights into the adsorption mechanism and may be more suitable for different systems [40].

The appropriate isotherm for describing Pt(IV) adsorption depends on the characteristics of the adsorbent material and the experimental conditions. For analyzing the experimental data, the nonlinear equations for each isotherm (Supplementary Material Table S1), as reported in the scientific literature, were applied [41,42,43].

The Taguchi method was employed to enhance the efficiency of the Pt(IV) removal process. An L16 orthogonal array experimental design (5 factors at 4 levels) was used to identify the optimal adsorption conditions, utilizing the “larger is better” criterion. Analysis of variance (ANOVA) with a general linear model was applied to calculate the percentage contribution of each factor to the Pt(IV) removal efficiency and to assess the results from the Taguchi method. The required mathematical calculations were performed using Minitab 19 software (version 19.1.1, Minitab LLC, State College, PA, USA).

### 2.4. Studies on the Recovery of Pt(IV) by Adsorption in Dynamic Regime

The aim of this experiment is to evaluate the efficiency of the biomaterial Chi–Ser in the adsorption-based recovery of Pt(IV) from aqueous solutions. By varying the height of the fixed bed in the column, the process is optimized, and the most effective bed height for Pt(IV) recovery is determined.

A 30 cm long and 2 cm diameter adsorption column was used, packed with varying amounts of biomaterial (10 g, 5 g, and 2.5 g), resulting in different material heights in the column (10 cm, 5 cm, and 2.5 cm). Aqueous Pt(IV) solution with an initial concentration of 100 mg/L was pumped through the column using a peristaltic pump (Heidolph SP quick, Heidolph Instruments GmbH & Co., Schwabach, Germany) at a constant flow rate of 3 mL/min. At regular intervals, 25 mL samples were collected to monitor the variation of the Pt(IV) concentration in the effluent. The residual Pt(IV) concentration was analyzed by atomic absorption spectrometry (AAS) using a Varian SpectrAA 280 FS spectrophotometer.

The experimental data will be used to determine adsorption isotherms, which describe the relationship between the concentration of Pt(IV) in the solid phase (adsorbed on the material) and the concentration in the liquid phase (in solution).

Evaluating the efficiency of an adsorbent material in a dynamic system requires an understanding of key process parameters, including the effluent flow rate, fixed-bed height, and contact time [44,45].

The Bohart–Adams, Clark, and Thomas models are widely used mathematical tools to describe and predict the behavior of adsorption systems over time. These models work by fitting experimental data and are crucial for understanding adsorption mechanisms as well as providing insights into the nature of the adsorption process (whether physical or chemical), the mass transfer rate, and the distribution of adsorbents in adsorption beds. For data analysis, linearized equations (Supplementary Material, Table S2) found in the scientific literature were employed [46,47,48,49].

### 2.5. Desorption and Reusability Studies

To assess the long-term performance of the Chi–Ser adsorbent material, repeated adsorption–desorption cycles were conducted. A glass column, 2 cm in diameter and 6 cm in height, was loaded with 10 g of material. A Pt(IV) solution with a concentration of 50 mg/L was passed through the column at a constant flow rate of 3 mL/min using a peristaltic pump (Heidolph Pumpdrive 5206, Heidolph Instruments GmbH & Co., Schwabach, Germany). 10 mL samples were collected at regular intervals, and the residual Pt(IV) concentration was determined using atomic absorption spectrometry. Multiple adsorption–desorption cycles were carried out until the material became exhausted, allowing for the determination of the maximum number of adsorption-desorption cycles. Desorption was performed using a 5% HCl solution.

## 3. Results and Discussion

### 3.1. Characterization of the Chi–Ser Material

Twenty-five adsorbent materials were synthesized by ultrasonic functionalization of chitosan (Chi) with five amino acids—aspartic acid (Asp), L-glutamic acid (Glu), valine (Val), DL-cysteine (Cys), and serine (Ser)—at chitosan-to-amino acid mass ratios of 1:0.05, 1:0.10, 1:0.15, 1:0.20, and 1:0.25. These materials were assessed for Pt(IV) adsorption under standardized conditions (initial concentration: 10 mg/L; contact time: 1 h; temperature: 298 K). The serine-functionalized chitosan demonstrated the highest adsorption capacity, ranging from 2.05 to 2.25 mg/g. Based on both performance and cost-effectiveness, a 1:0.10 mass ratio (Chi:Ser) was selected for further application, as higher ratios did not yield significant improvements.

**Figure 2:**
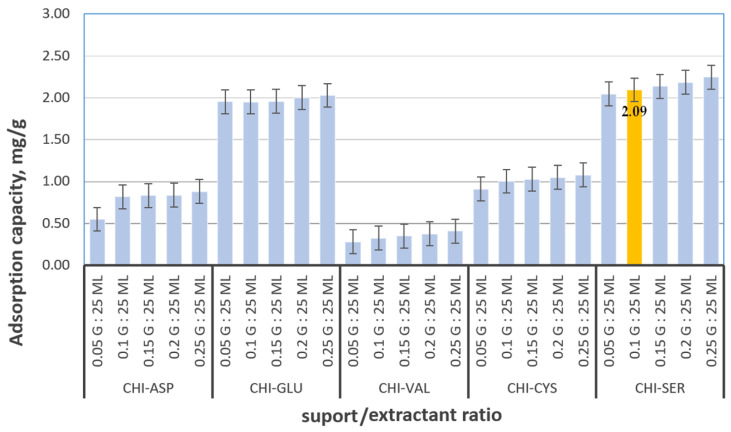
Material selection for Pt(IV) recovery by adsorption.

Figure 2 shows the comparative performance of the 25 synthesized materials in the Pt(IV) adsorption recovery process.

As illustrated in Figure 2, the amino acids employed for chitosan functionalization exhibit a pronounced affinity toward Pt(IV) ions, significantly enhancing the overall adsorption efficiency relative to unmodified chitosan, which demonstrated an experimentally determined adsorption capacity of 0.2 mg/g. Among all tested variants, the chitosan-serine combination achieved the best performance. While increasing the amino acid content generally improved adsorption, the 1:0.10 ratio was identified as optimal, balancing high adsorption capacity with economic feasibility.

The physicochemical characterization of the serine-functionalized chitosan material confirmed the successful attachment of serine, as evidenced by the presence of -NH_2_ and -COOH functional groups on the chitosan surface. This result validates the effectiveness of the functionalization process. The presence of these functional groups is essential for Pt(IV) ion adsorption, as they facilitate interactions through coordinate or ionic bonding, thereby enhancing adsorption efficiency.

To better understand the adsorption mechanism, the experimental findings will be correlated with the structural and chemical properties of the Chi–Ser material.

**Figure 3:**
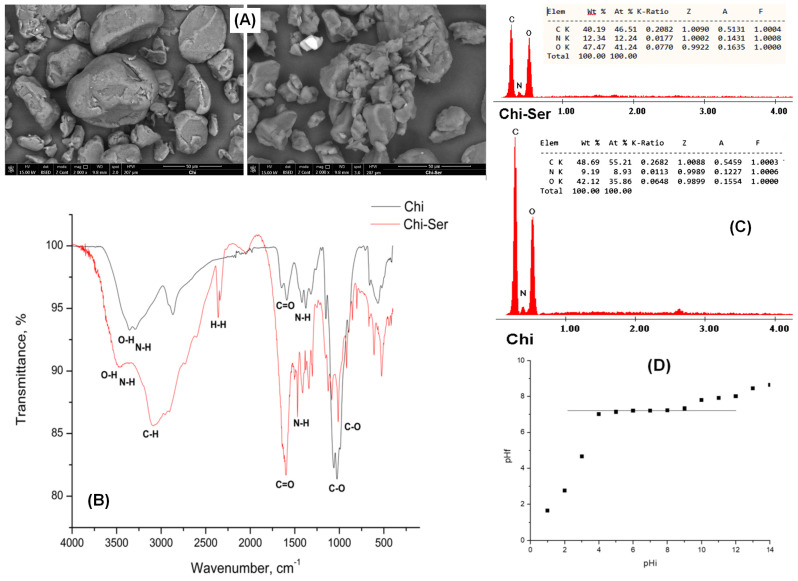
The physicochemical characterization of the Chi–Ser material: (**A**) scanning electron spectroscopy (SEM); (**B**) energy-dispersive X-Ray spectroscopy (EDX); (**C**) infrared spectroscopy (FT-IR); and (**D**) pH_PZC._

Figure 3 presents SEM micrographs, EDX spectra, FT-IR spectra, and the pH_PZC_ value of the Chi–Ser material.

The SEM image reveals that the functionalization of chitosan with serine resulted in notable modifications to the material’s surface morphology. These alterations can be attributed to interactions between chitosan and the amino acid, as well as potential structural modifications in chitosan following the functionalization process [50].

The EDX spectrum confirms the presence of nitrogen atoms on the chitosan surface. These nitrogen atoms originate from the amino (-NH_2_) groups of both serine and chitosan, verifying the successful attachment of serine to the chitosan structure [51]. The IR spectrum displays a broad band at 3474 cm^−1^, corresponding to the O–H stretching vibration characteristic of chitosan, as well as the N–H bending vibration associated with the –NH_2_ groups present in both chitosan and serine [52,53]. The absorption band at 3099 cm^−1^ is assigned to C–H stretching vibrations specific to serine [54], while the band at 2400 cm^−1^ is attributed to hydrogen bonding interactions characteristic of serine. A distinct peak at 1590 cm^−1^ corresponds to C=O stretching vibrations, common to both serine and chitosan. [55]. The C-O vibration for both chitosan and serine corresponds to the peak at around 980 cm^−1^ [56].

Controlling the electrical charge at the adsorbent–adsorbate interface is a key strategy for optimizing adsorption processes and developing materials with tailored properties. The point of zero charge (pH_PZC_) for the Chi–Ser material was determined to be 7.21. This value represents the pH at which the surface of the solid material carries an equal number of positive and negative charges, resulting in a net surface charge of zero [57,58]. At pH levels below 7.21, the surface of the solid support, as well as the amino acid structure, predominantly features protonated amine (-NH_2_) and carboxyl (-COOH) groups.

In the adsorption process, an excess of similarly charged chemical groups in the reaction medium generates electrostatic repulsion between the adsorbate and adsorbent, whereas oppositely charged groups create electrostatic attraction. These interactions significantly influence the interfacial dynamics between the solid support surface and the adsorbed species [59]. The presence of different functional groups on both the support and the amino acid can either enhance or hinder intermolecular interactions, thereby contributing to the stabilization or destabilization of the amino acid in an aqueous environment.

### 3.2. Pt(IV) Recovery Studies: Batch Adsorption Experiments

**Figure 4:**
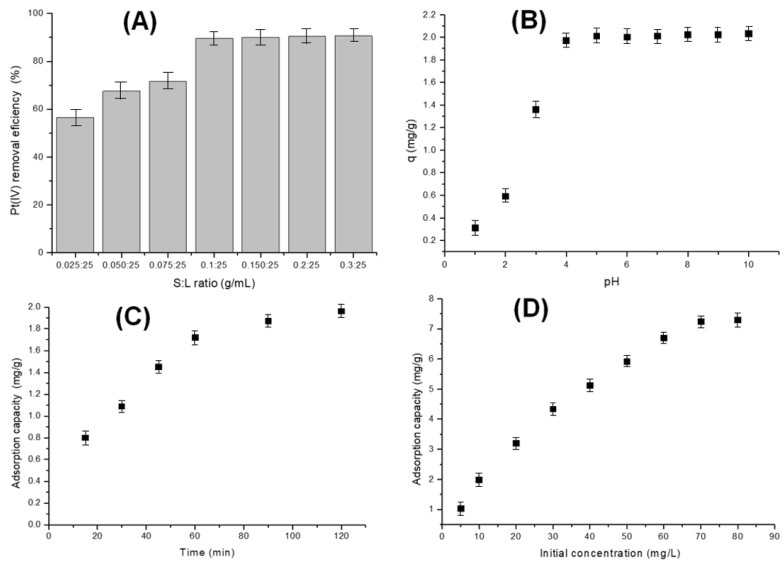
Influence of adsorbent dosage (**A**), pH (**B**), contact time (**C**), and initial Pt(IV) concentration (**D**) on adsorption efficiency.

Figure 4 shows the effect of dosages, pH, contact time, and initial concentration upon the Pt(IV) removal efficiency.

The effect of varying adsorbent dosages (0.025–0.3 g/25 mL) on Pt(IV) adsorption (10 mg/L) was evaluated through batch adsorption experiments. As shown in Figure 4A, Pt(IV) ions are rapidly adsorbed onto the bioadsorbent in the initial phase, reaching equilibrium over time. At an adsorbent-to-solution ratio of 0.1 g:25 mL with 10 mg/L Pt(IV), the adsorption efficiency reaches approximately 90%. However, equilibrium is attained after around 90 min (Figure 4C). Increasing the solid-to-liquid ratio beyond this point does not significantly enhance adsorption efficiency, even with extended contact time [60].

pH plays a critical role in adsorption processes, particularly for Pt(IV), as it can substantially impact both adsorption efficiency and mechanism. The speciation of Pt(IV) in a solution varies with pH, existing as cationic, neutral, or anionic species. In acidic conditions, Pt(IV) forms complex cationic species, whereas alkaline environments promote the formation of anionic species. These variations directly influence interactions with the adsorbent surface, affecting affinity for specific adsorption sites [61].

Chitosan’s chemical structure, which contains both hydroxyl and amino groups, makes it highly reactive for functionalization. However, in acidic solutions, amino groups are readily protonated [33]. This protonation enhances electrostatic attraction toward anionic species, including metal anions and anionic dyes. Additionally, the nitrogen atoms in chitosan possess lone electron pairs that can interact with metal ions, further contributing to its adsorption capabilities [62].

Figure 4B shows that the adsorption capacity of the Chi–Ser material remains constant at pH values above 4. A higher initial Pt(IV) concentration generates a stronger concentration gradient between the solution and the adsorbent surface. This gradient serves as the driving force for adsorption, leading to an increased uptake of Pt(IV) ions by the Chi–Ser material. At lower initial concentrations, ample free adsorption sites are available to accommodate all Pt(IV) ions present in the solution. However, as the initial concentration rises, these sites gradually become occupied, reducing the adsorption rate. Eventually, equilibrium is reached when all adsorption sites are saturated, with the maximum adsorption capacity of the material observed at 70 mg/L Pt(IV), corresponding to 7.23 mg/g. At very low initial concentrations, adsorption efficiency may decline due to excessive solution dilution. Conversely, at very high concentrations, the cost of effluent treatment can increase significantly, while the adsorption capacity of the material becomes a limiting factor [63].

Although the adsorption capacity of Chi–Ser is lower than that of other similar adsorbent materials—such as thiourea-modified magnetic biocarbons, Tu–N–SCG–C–A [1], L-lysine-modified crosslinked chitosan resin [27], glycine-modified crosslinked chitosan resin [38], and dibenzo-30-crown-10 ether immobilized on Amberlite XAD7 resin [64]—it offers several important advantages: (i) the reagents used in the synthesis of the material are environmentally friendly and relatively inexpensive; (ii) it can be used for the recovery of Pt(IV) at low concentrations (10 mg/L–80 mg/L) with good efficiency; and (iii) platinum with relatively high purity can be recovered through calcination, as the material contains only organic compounds.

**Figure 5:**
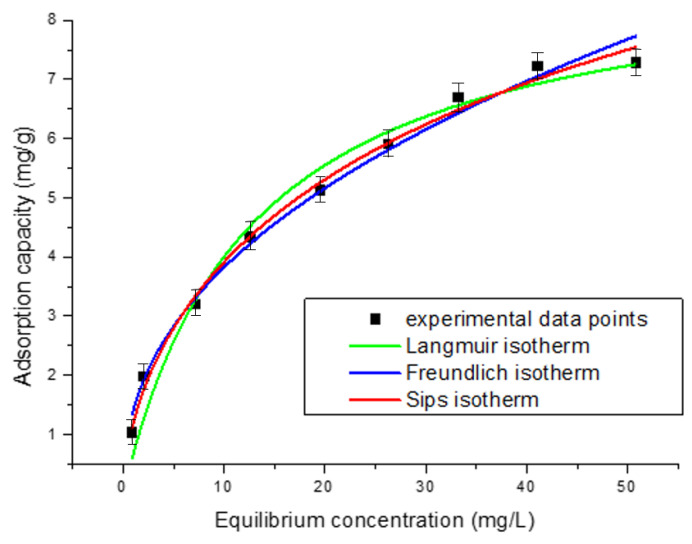
Equilibrium isotherms.

Figure 5 illustrates the relationship between the amount of adsorbed substance and its concentration in solution at equilibrium, as described by the Langmuir, Freundlich, and Sips adsorption isotherms. These isotherms provide a mathematical framework for modeling the adsorption process.

To analyze the interaction between the adsorbent and adsorbate, adsorption isotherms were applied. In this study, the Langmuir, Freundlich, and Sips isotherms were used at 298 K to evaluate Pt(IV) adsorption on the Chi–Ser material. As shown in Figure 5, the experimental values of qe increase progressively with higher initial Pt(IV) concentrations. The fitting results of the adsorption isotherm models, along with their derived parameters, are summarized in the Table 1. Based on the R^2^ and RMSE values, the Langmuir and Freundlich models exhibit lower correlations for Pt(IV) adsorption compared to the Sips model. These findings suggest that the adsorption of Pt(IV) on Chi–Ser follows the Sips model, indicating the possibility of multilayer adsorption.

### 3.3. Pt(IV) Recovery Through Fixed-Bed Column Studies

Key parameters for assessing the efficiency of an adsorbent material in a dynamic system include the effluent flow rate, the height of the fixed bed, and the contact time [1,33]. In the adsorption column, phenomena such as axial dispersion, external film resistance, and intraparticle diffusion resistance may occur. These processes are mathematically described by models that correlate axial dispersion, mass transfer, and intraparticle diffusion. To understand the adsorption mechanism of Pt(IV) and optimize the design of the dynamic adsorption process, it is essential to monitor the evolution of the residual effluent concentration over time. In this context, the Bohart–Adams, Thomas, and Clark models are commonly employed to analyze and predict the dynamic behavior of the column [34].

**Figure 6:**
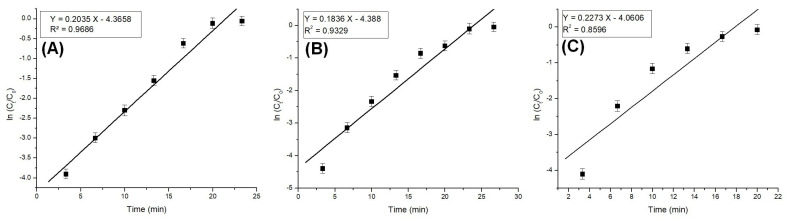
Bohart–Adams model plots for the adsorption of Pt (IV) at various amounts of adsorbent material; (**A**) 10 g; (**B**) 5 g; and (**C**) 2.5 g.

**Figure 7:**
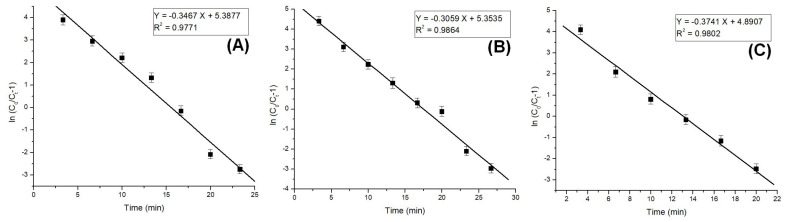
Thomas model plots for the adsorption of Pt (IV) at various amounts of adsorbent material; (**A**) 10 g; (**B**) 5 g; and (**C**) 2.5 g.

**Figure 8:**
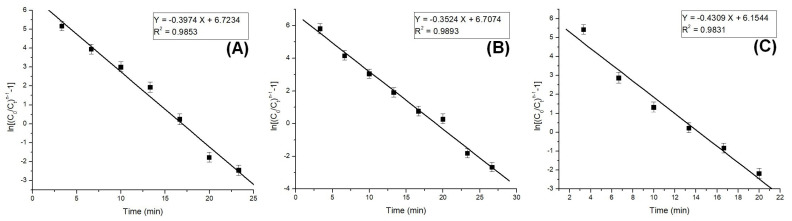
Clark model plots for the adsorption of Pt (IV) at various amounts of adsorbent material; (**A**) 10 g; (**B**) 5 g; and (**C**) 2.5 g.

The column model plots and corresponding parameters for all tested models in the dynamic adsorption process are shown in Figure 6, Figure 7 and Figure 8 and Table 2, respectively. It can be observed that all applied models reasonably fit the variation in the amount of adsorbed substance. The correlation coefficients for all models are high, indicating their validity in this study. Among the models, the Clark model demonstrates the highest values for the coefficient of determination (R^2^) and lower values for root mean square error (RMSE), regardless of the amount of adsorbent material used. Therefore, it can be concluded that the Clark model most accurately describes the adsorption process mechanism in a dynamic system.

### 3.4. Optimization of Adsorption Conditions in Batch System

The primary objective of the Taguchi method is to calculate and analyze the signal-to-noise (S/N) ratio to assess the quality of the experiment and the reliability of the results. The S/N ratio serves as an indicator of both variability and accuracy for each response observed in every trial. The “signal” represents the response influenced by each operational factor, while “noise” refers to any factors that affect precision. These elements are linked to the importance of the operational variables. A key advantage of this technique is its ability to reduce the number of experiments required while also providing insight into the optimal conditions. To maximize the efficiency of Pt(IV) removal, the “larger is better” option was applied for the S/N ratio [65,66]. Table 3 presents the controllable factors and their respective levels used in the Taguchi design, while Table 4 outlines the L16 orthogonal array, showing the controllable factors and the results obtained in each experiment conducted after each run.

The interaction effects of the various factors, based on the S/N ratio, along with the significance ranks of each factor, are presented in Table 5. The pH emerged as the most influential controllable factor on the process, while the S-L ratio had the least impact. By correlating the data from Table 5 with those in Table 3, the optimal conditions for the adsorption process can be determined: pH = 10, S-L ratio = 0.1, contact time = 90 min, temperature = 45 °C, and initial dye concentration = 5 mg/L. Additionally, the percentage contribution of each factor to the process efficiency, as derived from ANOVA analysis, is shown in Table 5.

### 3.5. Desorption and Reusability Studies

To assess the practical applicability, the potential for reusing the adsorbent material was examined through adsorption–desorption cycles.

**Figure 9:**
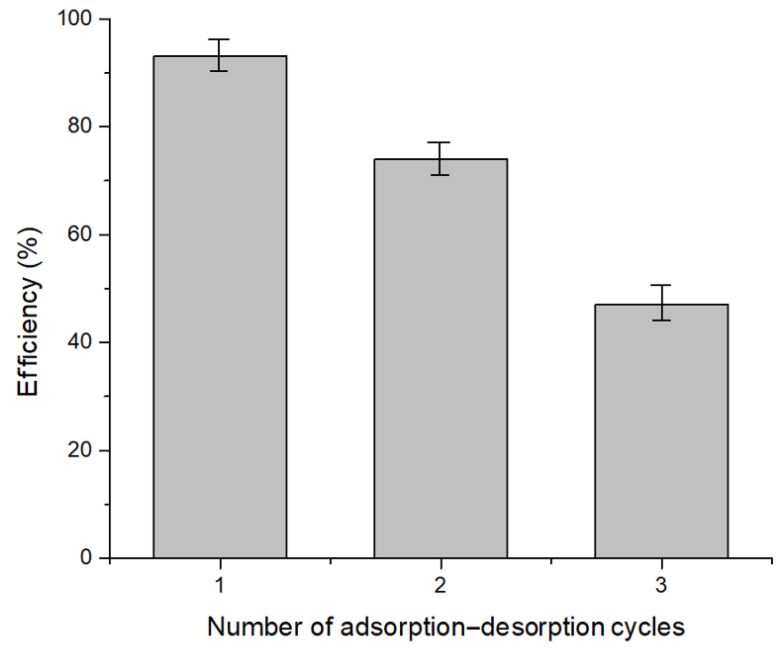
Studies on desorption and reusability.

As depicted in Figure 9, the recovery efficiency of Pt(IV) decreases with each successive adsorption–desorption cycle, dropping from 93% to 47% after three cycles. At a high chloride concentration and pH 2, Pt(IV) ions are primarily present in the forms [PtCl_6_]^2−^ and [PtCl_5_(H_2_O)]^−^, which enhances their affinity for the protonated functional groups on the Chi–Ser material. The desorption efficiency of the adsorbed Pt(IV) ions was evaluated using HCl.

## 4. Conclusions

In summary, the Chi–Ser material was synthesized by ultrasonic functionalization of chitosan with the amino acid serine, aiming to enhance the adsorption recovery of Pt(IV) from aqueous solutions. Adsorption is emerging as a promising technology for platinum recovery, offering a more selective and efficient alternative to conventional methods. However, ongoing research continues to focus on optimizing the adsorption process and developing new adsorbents.

SEM, EDX, and FT-IR analyses confirmed the successful functionalization of chitosan with serine, revealing significant changes in the surface properties of the material. These changes play a crucial role in enhancing the material’s performance during the adsorption of Pt(IV) ions.

The adsorption performance is primarily influenced by the preparation method of the adsorbent and experimental conditions, including adsorbent dosages, temperature, contact time, pH, and metal concentration. Notably, ultrasound-assisted activation offers a time- and energy-efficient approach for preparing adsorbents with the potential for large-scale applications.

By examining the effect of initial concentration on the adsorption process, optimal conditions for efficient Pt(IV) ion removal were identified. Understanding the concentration–adsorption relationship is vital for designing and sizing wastewater treatment systems dealing with heavy metal ions.

The initial concentration of Pt(IV) ions significantly affects the adsorption process, and comprehending this relationship enables the optimization of wastewater treatment methods and the development of more efficient adsorbent materials.

The adsorption recovery process in the static regime was best described by the Sips isotherm, while the Clark model provided the best fit for the dynamic regime.

Optimization of the static adsorption process using the Taguchi method revealed that pH has the greatest influence on the process, while the S-L ratio has the least impact.

Reusability studies indicated that after three adsorption–desorption cycles, the Chi–Ser material reaches a point of exhaustion, highlighting its limited lifespan for repeated use.

## Figures and Tables

**Table 1:** The tested isotherms models’ constants and the corresponding statistical parameters.

Isotherm Model	Parameters	Value
Langmuir	K_L_ (L/mg)	0.088 ± 0.011
	q_max_ (mg/g)	7.2 ± 0.7
	R^2^	0.9827
	RMSE	0.3336
Freundlich	K_f_ (mg/g)	1.41 ± 0.24
	1/n	0.43 ± 0.08
	R^2^	0.9788
	RMSE	0.2195
Sips	Q_sat_ (mg/g)	7.06 ± 0.94
	K_S_ (L/mg)	0.07 ± 0.01
	n	3.33 ± 0.43
	R^2^	0.9921
	RMSE	0.2154

**Table 2:** Pt(IV) adsorption process parameters on fixed-bed column.

Column Parameters					
Bohart–Adams model	Material amount (g)	K_B−A_ (L/mg·min)	N_0_ (mg/L)	R^2^	RMSE
	10	(20.30 ± 1.43) × 10^−4^	287.22 ± 17.32	0.9686	0.3799
	5	(18.36 ± 1.24) × 10^−4^	315.68 ± 24.42	0.9329	0.4340
	3	(22.73 ± 1.84) × 10^−4^	236.54 ± 11.41	0.8596	0.6406
Thomas model	Material amount (g)	K_Th_ (L/mg·min)	q_Th_ (mg/g)	R^2^	RMSE
	10	(34.67 ± 2.54) × 10^−4^	517.8 ± 27.53	0.9771	0.4177
	5	(30.59 ± 1.89) × 10^−4^	1165.1 ± 49.57	0.9864	0.3209
	3	(37.41 ± 3.02) × 10^−4^	1741.5 ± 61.54	0.9802	0.5246
Clark model	Material amount (g)	r (1/min)	A	R^2^	RMSE
	10	0.3974 ± 0.0478	831.64 ± 42.75	0.9853	0.2863
	5	0.3524 ± 0.0421	818.43 ± 35.74	0.9893	0.3161
	3	0.4309 ± 0.0624	470.78 ± 22.51	0.9831	0.3399

**Table 3:** The controllable factors and their levels, used in the Taguchi design.

Factor	Level 1	Level 2	Level 3	Level 4
pH	1	4	7	10
Solid/liquid ratio	0.025	0.1	0.2	0.3
Time (min)	15	50	90	120
Temperature (°C)	25	35	45	55
Initial dye concentration (mg/L)	5	25	50	80

**Table 4:** The L16 orthogonal array is constructed using the controllable factors and the results obtained from each experiment conducted after every run.

pH	S-L Ratio	Time (min)	Temperature (°C)	Initial Concentration (mg/L)	Efficiency (%)
1	0.025	15	25	5	4.23
1	0.1	50	35	25	13.6
1	0.2	90	45	50	14.8
1	0.3	120	55	80	2.25
4	0.025	50	45	80	14.0
4	0.1	15	55	50	18.4
4	0.2	120	25	25	59.2
4	0.3	90	35	5	99.2
7	0.025	90	55	25	21.6
7	0.1	120	45	5	99.6
7	0.2	15	35	80	8.87
7	0.3	50	25	50	37.4
10	0.025	120	35	50	23.4
10	0.1	90	25	80	35.5
10	0.2	50	55	5	99.4
10	0.3	15	45	25	31.6

**Table 5:** Response table for signal-to-noise ratios (larger-is-better option).

Level	pH	S-L Ratio	Time	Temperature	Initial Concentration
1	16.29	22.26	21.57	27.49	32.97
2	30.90	29.73	29.25	27.24	28.70
3	29.27	29.44	30.26	29.07	26.89
4	32.08	27.11	27.46	24.75	19.98
Delta	15.79	7.47	8.68	4.33	12.99
Rank	1	4	3	5	2
Contribution (%)	47.1	10.57	13.34	2.83	25.86

## Data Availability

All the experimental data are presented, in the form of tables and/or figures, in the article.

## Supplementary Materials

The following supporting information can be downloaded at https://www.mdpi.com/article/10.3390/polym17091132/s1, Table S1. The non-linear equations of the tested isotherms; Table S2. The linear equations of the tested column model.
