# Supplementary material for: Functionalized Biopolymer for Enhanced Pt(IV) Recovery from Aqueous Solutions
Source: Polymers (Basel). 2025 Apr 22;17(9):1132. doi: 10.3390/polym17091132 (PMC12073178; doi:10.3390/polym17091132)
Supplement: Supplementary file 1 [file polymers-17-01132-s001.zip › polymers-3590234-supplementary.pdf]

polymers

MDPI

Supplementary Material

Functionalized Biopolymer for Enhanced Pt(IV) Recovery from Aqueous Solutions

Theodora Babău [1], Mihaela Ciopec [1], Giannin Mosoarca [1,*], Cosmin Vancea [1,*], Adina Negrea [1], Nicoleta Sorina Nemeş [2], Bogdan Pascu [2], Petru Negrea [1,3], Catalin Ianăşi [4] and Alina Ramona Buzatu [5]

[1] Faculty of Chemical Engineering, Biotechnologies and Environmental Protection, Politehnica University Timisoara, Victoriei Square, no. 2, 300006 Timisoara, Romania; theodora.babau@student.upt.ro (T.B.); mihaela.ciopec@upt.ro (M.C.); adina.negrea@upt.ro (A.N.); petru.negrea@upt.ro (P.N.)
[2] Renewable Energy Research Institute-ICER, Politehnica University Timisoara, Gavril Musicescu Street, no. 138, 300774 Timisoara, Romania; nicoleta.nemes@upt.ro (N.S.N.); ioan.pascu@upt.ro (B.P.)
[3] ISIM-National R&D Institute for Welding and Material Testing, Timisoara, Bv. Mihai Viteazu, nr. 30, 300222 Timisoara, Romania
[4] "Coriolan Drăgulescu" Institute of Chemistry, Romanian Academy, Bv. Mihai Viteazu, No. 24, 300223 Timisoara, Romania; ianasic@acad-icht.tm.edu.ro
[5] Faculty of Medicine, "Victor Babes" University of Medicine and Pharmacy, Eftimie Murgu Square, no. 2, 300041 Timisoara, Romania; buzatu.ramona@umft.ro
* Correspondence: giannin.mosoarca@upt.ro (G.M.); cosmin.vancea@upt.ro (C.V.); Tel.: +40-256-404185 (G.M.); +40-256-404194 (C.V.)

Table S1. The non-linear equations of the tested isotherms.

Isotherm models	Equation
Langmuir isotherm	$q_e = \frac{q_m \cdot K_L \cdot C_e}{1 + K_L \cdot C_e}$
Freundlich isotherm	$q_e = K_F \cdot C_e^{1/n_F}$
Sips isotherm	$q_e = \frac{Q_{sat} \cdot K_S \cdot C_e^n}{1 + K_S \cdot C_e^n}$

where: q_m and Q_{sat} represents the maximum absorption capacities; K_L, K_F and K_S represents the Langmuir, Freundlich and Sips isotherms constants; $1/n_F$ represents an empirical constant indicating the intensity of adsorption; n represents Sips isotherm exponent.

Table S2. The linear equations of the tested column model.

Column models	Equation
Bohart–Adams model	$\ln\left(\frac{C_t}{C_0}\right) = k_{BA} C_0 t - k_{BA} N_0 \frac{Z}{F}$
Thomas model	$\ln\left(\frac{C_0}{C_t} - 1\right) = \frac{k_{Th} q_{Th} m}{Q} - k_{Th} C_0 t$
Clark model	$\ln\left(\left(\frac{C_0}{C_t}\right)^{n-1} - 1\right) = \ln A - rt$

where: C_0 represents the influent concentration; C_t represents the effluent concentration; t represents time; k_{BA} represents the kinetic constant of the Bohart-Adam model; F represents the linear velocity calculated by dividing the flow rate by the column section area; Z represents the bed height of column; N_0 represents the saturation concentration; k_{Th} represents the Thomas rate constant; q_{Th} represents the equilibrium compounds uptake per g of the resin; m represents the mass of adsorbent resin; Q represents he flow rate; n

Academic Editor: Firstname Lastname

Received: date
Revised: date
Accepted: date
Published: date

Citation: To be added by editorial staff during production.

Copyright: © 2025 by the authors. Submitted for possible open access publication under the terms and conditions of the Creative Commons Attribution (CC BY) license (https://creativecommons.org/licenses/by/4.0/).

represents the Freundlich constant determined experimentally in batch; r represents the Clark model constant; A represents the Clark model constant.
